# The Autophagy-Cilia Axis: An Intricate Relationship

**DOI:** 10.3390/cells8080905

**Published:** 2019-08-15

**Authors:** Manuela Morleo, Brunella Franco

**Affiliations:** 1 Telethon Institute of Genetics and Medicine (TIGEM), Via Campi Flegrei, 34, 80078 Pozzuoli, Naples, Italy; 2 Medical Genetics, Department of Translational Medical Sciences, Federico II University of Naples, Via Sergio Pansini 5, 80131 Naples, Italy

**Keywords:** primary cilium, autophagy, Hedgehog signaling, ciliopathy

## Abstract

Primary cilia are microtubule-based organelles protruding from the surface of almost all vertebrate cells. This organelle represents the cell’s antenna which acts as a communication hub to transfer extracellular signals into intracellular responses during development and in tissue homeostasis. Recently, it has been shown that loss of cilia negatively regulates autophagy, the main catabolic route of the cell, probably utilizing the autophagic machinery localized at the peri-ciliary compartment. On the other side, autophagy influences ciliogenesis in a context-dependent manner, possibly to ensure that the sensing organelle is properly formed in a feedback loop model. In this review we discuss the recent literature and propose that the autophagic machinery and the ciliary proteins are functionally strictly related to control both autophagy and ciliogenesis. Moreover, we report examples of diseases associated with autophagic defects which cause cilia abnormalities, and propose and discuss the hypothesis that, at least some of the clinical manifestations observed in human diseases associated to ciliary disfunction may be the result of a perturbed autophagy.

## 1. Introduction

Cilia are microtubule-based organelles protruding from the cell surface of almost all mammalian cells and exert diverse motility and sensory functions. They consist of an axoneme of nine microtubule doublets anchored by a basal body, which is derived from the mother centriole of the centrosome, and a transition zone functioning as a gate that strictly controls the protein composition of ciliary compartments. Cilia assembly and maintenance rely on the intraflagellar transport (IFT) machinery that uses kinesin and dynein motors to transport cargoes from the cell body to the ciliary tip and back. Cilia can be broadly subdivided into two groups. Motile cilia, which are multiple protrusions on the surface of epithelial cells involved in moving fluids such as tracheal and neuronal ependymal cells, and primary cilia, which appear as non-motile single organelles with sensory functions. For a detailed description of cilia structure, formation, assembly, and maintenance please refer to [1]. Ciliary dysfunction has been implicated in disorders called “ciliopathies” which present overlapping phenotypes, such as primary ciliary dyskinesia, retinal degeneration, renal, hepatic and pancreatic cysts, skeletal defects, situs inversus, obesity, mental retardation, and CNS malformations. Examples of ciliopathies include Bardet–Biedl syndromes (BBS), oral-facial-digital type 1 syndrome (OFDI), autosomal dominant and autosomal recessive polycystic kidney diseases (ADPKD and ARPKD, respectively), Joubert syndrome (JS) and related disorders, nephronophthisis, Meckel Grouber and Birt-Hogg-Dube’(BHD) syndromes [1]. Moreover, a number of evidence recently showed that cilia formation is compromised in multiple human cancers including pancreatic cancer, melanoma, breast cancer, cholangiocarcinoma, renal cell cancer, and Hurthle cell carcinoma [2,3].

Primary cilia are complex sensory machines involved in transducing extracellular stimuli into cellular responses and are used by the majority of cells to maximize sensory functions and respond to environmental inputs [4]. Signal transduction in the cilium coordinates key processes during development, tissue maintenance, and regeneration (reviewed in [5]). The sensory modalities to which cilia respond include chemosensation (response to stimuli given by specific ligands, growth factors or hormones) and mechanical stimulation (bending of the cilium) depending on the cell type. In specialized cells, primary cilia can respond to insulin receptor-mediated stimuli [6], to light [7], temperature [8], osmolarity [9], or gravity [10]. A single primary cilium can be used by diverse signaling pathways, such as Wnt, Notch, PCP, Pdgfα, and the Hedgehog (Hh) since different receptors or channels can coexist and exert their function on the same cilium [11].

One of the most characterized cilia-dependent paracrine signaling is the Hh pathway, which plays an essential role in promoting development and tissue homeostasis, as well as in regulating cell fate, cancer and stem cells renewal [12,13]. Hh, unlike other signaling pathways, is fully dependent on primary cilia (reviewed in [14]). In vertebrates, the Hh ligands, which in mammals include Sonic (Shh), Indian (Ihh), and Desert (Dhh), can initiate signaling upon binding to the receptor, Patched on the cilium. The subsequent stimulation of the ciliary membrane protein Smoothened (Smo) leads to the activation of Gli proteins, the transcriptional effectors of the pathway, that are localized at the tip of the cilium [14].

Recent studies showed that primary cilia, ciliary signaling pathways, and ciliary proteins control macroautophagy, (hereafter autophagy) and that, conversely, autophagy is one of the main players in the regulation of ciliogenesis [15]. Autophagy is one of the main paths that the cells use for degrading intracellular proteins and organelles, to achieve balancing of energy sources in development and in response to nutrient stress. As such, autophagy is a fundamental biological process with a key role in normal development, adult tissues homeostasis, and in pathological conditions such as neurodegenerative diseases, muscle and liver diseases, and cancer [16]. Under nutrient depletion, a number of autophagy related proteins (ATGs) orchestrate the formation of autophagosomes, which are the membranous organelles instrumental for autophagy. Fusion of autophagosomes with lysosomes results in degradation of the autophagosomes content. Among several ATG core proteins, the ATG8 conjugation system, which comprises the microtubule-associated protein 1 light chain 3 (MAP1LC3, hereafter referred to as LC3), γ-aminobutyric acid receptor-associated protein (GABARAP), and Golgi-associated ATPase enhancer of 16 kDa (GATE-16), is required for autophagosome elongation and maturation [17]. A number of upstream signaling complexes regulate autophagy, including the pathway of mTOR, a serine–threonine kinase which positively regulates cell growth and proliferation by promoting anabolic activities, and by limiting catabolic processes such as autophagy [18]. The macro-autophagic response to starvation involves the bulk degradation of cytosolic material, whereas selective autophagy involves recognition and removal of specific targets. The selectivity is achieved through autophagy receptors and adaptors, such as p62, NBR1, and NDP52, which recognize cargoes and the autophagosomal membrane through their LC3-interacting regions (LIR) (reviewed in [19]). The receptors sequester cargoes and deliver them to lysosomes where cargoes and receptors are both degraded [20,21].

In this review we discuss recent findings which uncovered the dual crosstalk between cilia and autophagy and the direct functional interplay occurring between the core autophagic machinery and at least some of the ciliary proteins. We propose that this functional relationship can modulate both ciliogenesis and autophagy to ensure a feedback loop. Moreover, we discuss recent discoveries regarding the role of the cilia-autophagy link in the pathogenesis of some of the clinical manifestations observed in cilium- and autophagy-related disorders.

## 2. The Autophagy Machinery Localizes at Cilia

Pampliega and colleagues described in 2013 that autophagy-related proteins localize at cilia or periciliary regions. Indeed they showed that, staining of both mature autophagosome markers (i.e., LC3 and GABARAP) and autophagy proteins acting on the initial steps of autophagosome formation (i.e., VPS15, ATG16L, and AMBRA1), showed discrete puncta at basal bodies and cilia axonemes [22,23]. VPS34, VPS14, and autophagosome elongation complex players such as ATG7 and ATG5, were instead found at the basal body [23]. The authors propose that the ciliary membrane could act as a new nucleation site for pre-autophagosome formation that uses the plasma membrane as a source of autophagosome [24], despite most of the early autophagic processes are found to take place near the endoplasmic reticulum or the outer mitochondrial membranes [25,26]. However, Beclin 1, a key player acting during the initiation stage of autophagy through the formation of the isolation membrane, is not localized at cilia, suggesting the existence of different autophagosome initiation complexes.

**Figure 1:**
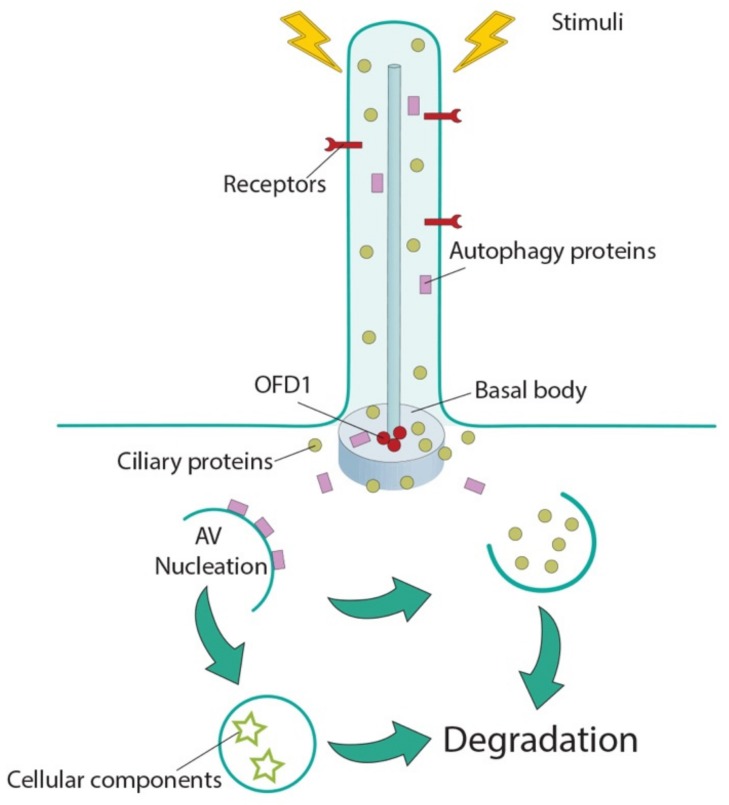
Cilia-autophagy crosstalk. Cilia sense external stimuli and transduce them into the cell to influence macroautophagy, possibly utilizing the autophagic machinery localized in the peri-ciliary compartment. On the other side, autophagosomes influence ciliogenesis by selective autophagic degradation of ciliary positive and/or negative effectors as cargoes. AV: autophagic vacuoles; ciliary proteins: green circles; OFD1: red circles; autophagic proteins: purple rectangles; cellular components: stars.

The finding that the autophagy core machinery is localized at ciliary compartments suggests that: (a) cilia may represent a strategic site to ensure smoothest and fast activation of autophagy in response to the different stimuli coopted by the sensorial antenna of the cell (Figure 1); and that (b) selective autophagic degradation may remove positive and/or negative regulators of ciliogenesis, thus influencing cilia formation and elongation (Figure 1).

However, the molecular mechanisms underlying the direct role of core autophagic proteins in cilia biology, and, conversely, the impact of this sensing organelle and of ciliary-mediated signaling pathways in autophagy remain elusive. Very recently, direct functional interactions between cilioproteins and core autophagic proteins have emerged, suggesting far-reaching implications for cilium-and autophagy-related disorders (see below).

## 3. Autophagy Controls Ciliogenesis and Cilia Length

Contrary to most mammalian cells in vivo, the majority of in vitro cultured cells are not ciliated: primary cilia are assembled when cells exit cell cycle to enter quiescence [4]. Early studies showed that serum deprivation promotes cell cycle arrest inducing ciliogenesis in cultured cells [27]. Interestingly, the removal of serum promotes autophagy [28]. Despite common stimuli promotes both ciliogenesis and autophagy, these have been largely seen as independent cellular processes. In 2013 Tang and colleagues were the first to demonstrate a positive role for serum deprivation induced-autophagy in ciliogenesis. However, other laboratories reported an opposite function for induced autophagy in cilia elongation [22,29,30], suggesting that the role played by this degradative pathway might depend on the cellular context (see below).

Tang et al. showed that autophagy promotes ciliogenesis through selective degradation of OFD1, a ciliopathy protein localized both to the distal end of centrioles and to centriolar satellites; the OFD1 centriole population is essential for ciliogenesis, while the satellite pool acts as a ciliogenesis suppressor. Autophagy specifically degrades the satellite pool of OFD1 to promote cilia formation in retinal pigmented epithelial (RPE) cells and mouse embryonic fibroblasts (MEFs) [31]. The authors also showed that tandem affinity purification of LC3 recovered the centriolar satellites proteins PCM1, OFD1, and CEP131 and that LC3 specifically targets OFD1 to autophagosomes for degradation, leaving other centriolar satellites markers and the basal body portion of OFD1 unchanged [31]. These findings suggest that LC3 could use OFD1 as an autophagy adaptor or receptor for selective, still unidentified, cargoes, which need to be removed by autophagy to promote ciliogenesis. To validate this hypothesis, studies exploring the interaction of OFD1 with LC3 and other OFD1-interacting proteins will be needed to identify and characterize the potential substrate/s of the autophagy pathway that uses OFD1 as an adaptor/receptor.

Conversely, however, in the same issue of *Nature*, Pampliega and colleagues demonstrated, in non-ciliated cycling wild type MEFs, that basal autophagy acts as a negative regulator of ciliogenesis by degrading IFT20 [22], a protein essential for both cilium formation and assembly [32]. Thus, the two studies describe two different and complementary roles of autophagy in the regulation of ciliogenesis, by selectively eliminating either (a) IFT20 during cell growth and proliferation (thus impairing ciliogenesis), or (b) the satellite pool of OFD1 during growth arrest (thus promoting ciliogenesis). The mechanisms underlying selective autophagic degradation of positive and negative effectors of ciliogenesis is still to be determined and could involve specific autophagy receptors which selectively recognize ciliary cargoes to promote or inhibit ciliogenesis. In addition, post-translational modifications, such as ubiquitination, acetylation, and phosphorylation of ciliary proteins acting as autophagy substrates, could be crucial for cargoes recognition and physical interaction with autophagy receptors [33]. Evidences that different cilia components are substrates for autophagic degradation support this hypothesis [22,30,31,34]. In addition to the abovementioned manuscripts, more recent studies also confirmed that autophagy promotes ciliogenesis. Liu and colleagues in 2018 reported that PPARA and NR1H4, known as nutrient-sensing receptors, influence ciliogenesis via a mechanism that controls expression of autophagy genes in different cell types (RPE, MEFs, human kidney-2 (HK2)) [35]. In particular, PPARA induces ciliogenesis in fasting condition, while NR1H4 negatively regulates cilia formation under nutrients [35]. In addition, it has been shown that autophagy activation also promotes cilia elongation [31,36], and conversely autophagy inhibition decreases cilia length in HK2 cells [36]. Another example is given by specific compounds: (1) sertraline, an antidepressant of a selective serotonin reuptake inhibitor class and (2) thioridazine, member of the phenothiazine family used as antipsychotic. Both drugs induce ciliogenesis through autophagy activation in RPE cells and in human lung cancer cells (A549) [37,38]. 

In contrast to the findings described above, Pampliega et al. found that autophagy is not required for cilia formation, since Atg5^−/−^ MEFs formed longer cilia compared to controls upon serum removal [22]. Struchtrup and colleagues later on showed that inhibition of autophagy via 3-methyladenine (an inhibitor of class III PtdIns3K and thus of early stages of autophagy) leads to increased cilia length in wild type MEFs and, conversely, activation of autophagy by rapamycin (an mTOR inhibitor) or ABT-737 (that affects autophagy but not protein synthesis) reduces cilia length in the same cells. Interestingly, they also showed that MEFs depleted for Rpgrip1l, a protein that localizes at the ciliary transition zone, have reduced autophagic activity which leads to elongation of cilia [29]. On the same line, Lam and colleagues demonstrated that chronic exposure to cigarette smoke leads to reduction of motile cilia length in epithelial cells, because of increased autophagy. The authors propose the term “ciliophagy” for the autophagy-dependent mechanism through which smoke induces cilia shortening [30]. These findings suggest that autophagic degradation could exert an opposite role on cilia biology in a context-dependent manner and that a number of autophagic players and ciliary cargoes could be involved according to the different cell type.

Moreover, it has been previously reported that selective degradation of ciliary proteins can also be mediated by the ubiquitin-proteasome system (UPS) to control ciliogenesis and cilia maintenance [39,40]. Autophagy and UPS are, at least partially, redundant and a crosstalk exists between them concerning their role on cilia biology [40], suggesting that the mechanism/s underlying degradation of positive and negative effectors of ciliogenesis could be intricate and depending on different cell types and/or culture conditions.

The finding that autophagy influences ciliogenesis could indicate the need of cells to generate a feedback mechanism to ensure formation of the sensing organelle committed to control the main degradative route of the cell (Figure 1). This implies that abnormal autophagy could underlie some of the clinical manifestations observed in ciliopathies and that abnormal cilia formation/function could be associated with autophagy-related disorders. Further studies on selective degradation of ciliary proteins will be necessary to investigate such molecular mechanisms which can contribute to the design of therapies for human conditions associated with ciliary dysfunction.

## 4. Cilia Control Autophagy

Emerging evidence also demonstrated that cilia are regulators of autophagy [22,23]. Indeed, it has been shown that autophagy activation upon serum starvation requires the presence of a functional primary cilium as IFT20-and IFT88-inactivated cells (MEFs and kidney epithelial cells (KECs)) show defective ciliogenesis and decreased autophagy upon serum removal. Moreover, the mTOR inhibitor rapamycin fails to restore normal autophagic activity in the same cells [22]. On the same line, Wang and colleagues showed a role for cilia in autophagy using two different renal cellular systems: IFT88-inactivated HK2 cells showing shorter cilia, and wild type renal epithelial cells selected for the presence of shorter cilia. In both cases the authors confirmed that cells characterized by shorter cilia display autophagy inhibition because of mTOR activation [36]. Moreover, Orhon and colleagues showed that in in vitro and in vivo renal systems, fluid flow induces autophagy which in turn regulates cell-volume and that this process is orchestrated by signal transduction pathways depending on intact and properly functioning primary cilia [41,42]. Another piece of evidence is provided by Jang and colleagues as they demonstrated that inhibition of cilia-mediated autophagy blocks proper neuroectodermal differentiation confirming the functional role of cilia in autophagy regulation [43]. Table 1 summarizes the ciliary proteins involved in impaired cilia-mediated autophagy in different cells types.

The involvement of a sensory platform as the primary cilium in the regulation of autophagy suggests that multiple stimuli coopted by the cilium in each cell, (e.g., changes in paracrine stimuli, fluid flow, light, pressure, temperature, as well as sensing of the nutritional status of the whole organism by the insulin system) could affect this catabolic process in developmental and adult stages in a cilia-dependent manner. Further studies will be needed to verify this hypothesis.

## 5. Hedgehog Signaling Is a Regulator of Autophagy

The Hh signaling is a regulator of autophagy, although contrasting evidence have been reported concerning the crosstalk between this signaling pathway and autophagy.

Stimulation of Hh in MEFs and KECs activates the expression of Hh target genes and concomitantly induces autophagy [22]. This is true also in two additional experimental conditions in which Hh signaling is activated: Patched knockout cells in which the Hh signaling is constitutively activated, and GLI1 overexpressing cells [22]. Conversely, silencing of SMO and treatment with Hh antagonist cyclopamine reduce autophagy [22]. In addition, Hh stimuli are unable to induce autophagy in cells with defective ciliogenesis, while GLI1 overexpression partially rescues autophagic defects in ciliary defective cells [22]. In line with these findings, Shh stimuli induce autophagy in hippocampal neurons through increased expression of autophagy-related genes and the authors suggest that this enhanced autophagy could have a role in presynaptic differentiation of hippocampal neurons [53]. Furthermore, it has been shown that Shh induces autophagy also in murine and human smooth muscular cells, through activation of AKT and independently of the mTOR pathway, to control the development of blood vessels [54].

However, discrepancies on the response of autophagy induction to Hh signaling activity have been reported. Jimenez-Sanchez and colleagues found that Hh signaling impairs autophagosome biogenesis in HeLa cells and MEFs as well as in *Drosophila*. The authors demonstrated that activation of the canonical Hh pathway through Gli2 is necessary for autophagy inhibition [55]. Findings from Tsai et al. confirmed enhanced autophagy in Gli2^−/−^ NIH3T3 fibroblasts, possibly because of decreased Gli transcriptional activities [56]. In addition, it has been recently described that the Hh pathway suppresses autophagy levels to control osteoblast differentiation in *zebrafish larvae* and, in particular, Gli2 depletion increases autophagy through enhanced protein levels of ATG5 and LC3 [57]. Finally, studies in human hepatocellular carcinoma and in pancreatic ductal adenocarcinoma cells suggest that inhibition of Hh signaling induces autophagy by modulating a number of biological functions [58,59,60]. The contradictory data could have resulted from the possible different roles of the Hh pathway on autophagy depending on the type of cells used in the studies, whether they are ciliated or not, on the conditions of the Hh pathway activation and of autophagy induction.

## 6. The Direct Functional Interplay between Ciliary and Core Autophagic Proteins

The bidirectional relationship between autophagy and cilia is deep and intricate, however, the main players coordinating this crosstalk as well as their functional roles remain unknown. We propose a novel interpretation that can pave the way to dissect the molecular mechanisms underlying this biological process. Ciliary proteins can be regarded as novel noncanonical autophagic players, which control (a) macroautophagy, independently from their role in ciliogenesis and (b) selective autophagic degradation of positive and/or negative effectors of ciliogenesis with the final aim of controlling ciliogenesis.

The first example of a ciliary protein directly involved in the regulation of starvation-induced autophagy was described in 2013. IFT20, the IFT protein involved in the trafficking of ciliary membrane proteins from Golgi to the base of cilia [32], physically interacts and colocalizes with ATG16L, and promotes its shuttling from Golgi-to-cilia during serum starvation through an IFT88-dependent mechanism [22]. These findings support a role for IFT proteins in the relocation of the autophagic machinery to cilia [22]. Subsequently a number of reports (described below) showed that proteins localized at cilia and controlling ciliogenesis display a direct functional role in the regulation of autophagy in non-ciliated conditions. Table 1 describes cilioproteins implicated in the control of autophagy independently from their roles in ciliogenesis.

PCM1 is a structural protein of centriolar satellites involved in ciliogenesis [61,62]. PCM1 physically interacts with GABARAP through a LIR motif and controls GABARAP localization and degradation at peripheral centriolar satellites thus influencing the GABARAP-autophagosome formation [48]. In the same paper it was also shown that PCM1 colocalizes with early autophagosome markers. The experiments were performed in non-ciliated conditions, leading the authors to hypothesize that the role of GABARAP-PCM1 on autophagosome biogenesis is independent from cilia [48].

Furthermore, Hasegawa and colleagues showed that an inositol 5-phosphatase, INPP5E, which is codified by one of the genes mutated in Joubert syndrome (JS) [63], is a positive regulator of autophagy [47]. INPP5E localizes at primary cilia, and its inactivation results in shorter cilia [63] and suppression of cilia-mediated Hh signaling [64,65]. Hasegawa et al. demonstrated that INPP5E localizes also at lysosomes and is required for autophagosome–lysosome fusion [47]. Despite the role of INPP5E in ciliogenesis, the authors performed all experiments in non-ciliated neuronal cells suggesting that the role of INPP5E in autophagosome–lysosome fusion is cilia-independent [47]. In addition, they showed that INPP5E mutations, affecting the phosphatase activity of the enzyme, are associated with impaired autophagy [47]. Future studies will determine whether autophagy defects could underlie some of the clinical manifestations observed in JS, and whether other cilioproteins mutated in this condition (34 to date) could have a role in this catabolic process.

Interestingly, a second inositol 5-phosphatase (PI(4,5)P2 5-phosphatase) known as OCRL1, localized at basal bodies and along axonemes, and with a role in cilia biogenesis [66], plays a direct functional role in autophagy [49]. OCRL is mutated in Lowe syndrome and Dent-2 disease, rare X-linked conditions [67] in which patients fibroblasts display shorter cilia [66,68,69]. De Leo et al. demonstrated that also OCRL is recruited by lysosomes and is required for autophagosome–lysosome fusion [49], and that loss of catalytic activity of OCRL causes accumulation of autophagosomes and lysosomal anomalies in cells isolated from Lowe syndrome patients [49].

Another example of ciliary protein directly involved in autophagy control is given by Folliculin (FLCN) which is localized at primary cilia and when inactivated, results in impaired ciliogenesis [70]. Mutations in the *FLCN* gene are responsible for Birt-Hogg-Dube’(BHD) syndrome [71]. Changes in FLCN levels are associated with dysregulation of Wnt and PCP signaling pathways [70], which are transduced through cilia. FLCN localizes also at lysosomes and modulates nutrient sensing by acting as a GTPase activating protein for RagC/D GTPases that signal amino acid levels to mTOR kinase [51]. Moreover, Dunlop and colleagues showed that FLCN physically interacts with the components of the autophagic machinery (e.g., GABARAP and ULK1 kinase), playing a positive role in autophagy [50]. Finally, kidney samples from BDH patients show autophagic defects [50].

In addition, Huntingtin (HTT), which is mutated in inherited Huntington neurodegenerative disease, has been found to be located at centrosomes and mediates the transport of PCM1 between cytoplasm and pericentriolar material [72,73]. When HTT is mutated, PCM1 accumulates, thus leading to increased ciliogenesis [72,73]. In addition to its ciliary role, HTT has been shown to physically interact with two main core regulators of autophagy: with p62 to facilitate its association with LC3 and its cargoes, and with ULK1 kinase, to influence selective autophagy [52]. 

Finally, the first example of an autophagic protein directly involved in ciliogenesis comes from VPS15, which encodes for a regulatory subunit of the class III phosphatidylinositol 3-phosphate lipid kinase VPS34/PIK3C3. VPS15 in association with VPS34 is involved in two well-studied protein modules, the UVRAG/Beclin1 and the Atg14L/Beclin1 complexes required for membrane trafficking and autophagy, respectively [45,46]. VPS15 localizes to cilia (basal bodies and axonemes) [22] and fibroblasts from patients with a mutation in the *VPS15* gene displaying a ciliopathy phenotype (retinitis pigmentosa, limb abnormalities and renal cysts) show shorter cilia, because of defective formation and/or release of IFT20 positive vesicles from the cis-Golgi [44].

**Figure 2:**
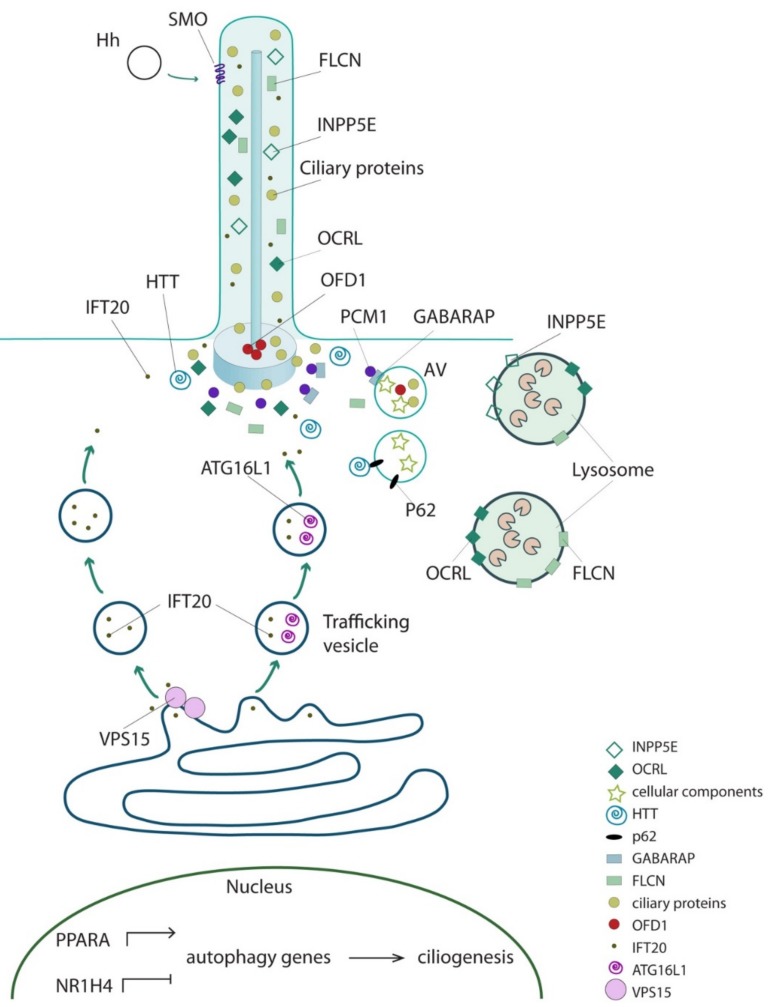
The interplay between ciliary and core autophagic proteins. Schematic representation of subcellular localization of cilioproteins exerting a direct functional role in autophagy. AV: autophagic vacuoles.

The examples listed above suggest that a variety of proteins localizing at cilia, with a role in cilia formation and maintenance, and/or associated with ciliopathies, share a direct functional role in autophagy independent from their role in cilia biology. We hypothesize that additional cilioproteins could have a direct functional role in different stages of the macroautophagic cascade (e.g., autophagy induction, autophagosomes nucleation, expansion and fusion with lysosomes) and could coordinate ciliogenesis using selective autophagy as a feedback loop in ciliated conditions. Moreover, we propose that cilioproteins could have both a positive and/or a negative effect on autophagy, since autophagic degradation could exert an opposite role on ciliogenesis in a context dependent manner. Finally, autophagy-independent ciliary functions for proteins involved in autophagic processes, such as the case of VPS15, cannot be ruled out. Figure 2 schematizes the subcellular localization of known cilioproteins exerting a direct functional role in autophagy.

These observations suggest that ciliary proteins might be considered as novel noncanonical autophagic players. The open question to be answered is whether they may concomitantly have a structural role in cilia biology and a regulative role on selective autophagic degradation with the final aim of controlling ciliogenesis. Further understanding of the molecular mechanisms underlying these functional interactions will be of the outmost interest not only from a basic science point of view but also for the possible therapeutic implications in ciliopathies, cancer, and neurodegenerative diseases.

## 7. Autophagy-Cilia Crosstalk in Disease

**Figure 3:**
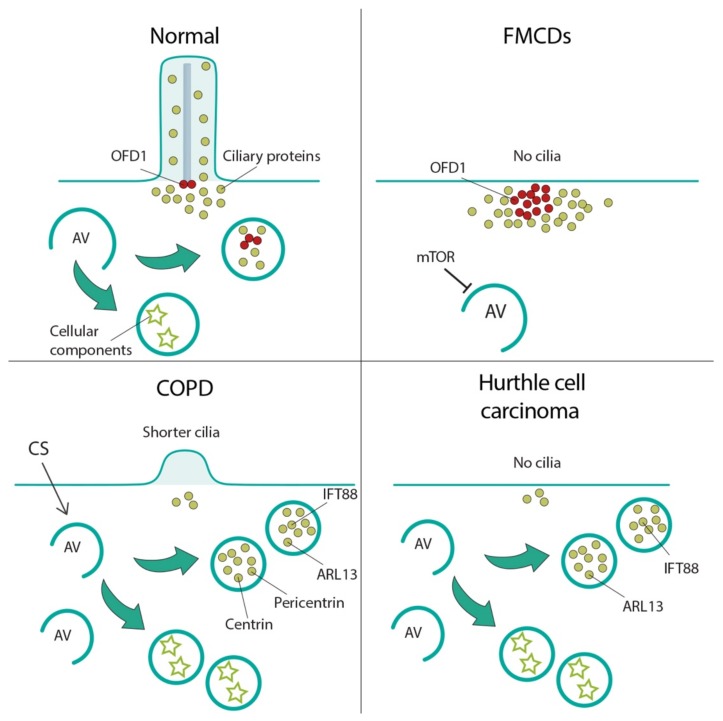
Cilia-autophagy crosstalk in diseases. Schematic representation of diseases characterized by cilia abnormalities because of autophagic defects. Focal cortical dyslamination (FMCDs), top right; in FMCDs neurons autophagy is inhibited by mTOR overactivation, which results in loss of autophagic degradation of the satellites pool of the OFD1 protein, a ciliogenesis inhibitor, with consequent defective formation of cilia. Chronic obstructive pulmonary disease (COPD), bottom left; autophagy increases in COPD cells under cigarette smoke (CS) exposure and causes cilia shortening by increased autophagic degradation of ciliogenic proteins. Hurthle cell carcinoma, bottom right; high basal level of autophagic activity suppresses ciliogenesis by persistent sequestration of ciliogenic proteins into autophagosomes. AV: autophagic vacuoles; ciliary proteins: green circles; OFD1: red circles; autophagy proteins: purple rectangles; cellular components: stars.

Very recent studies reported examples of diseases because of either increased or decreased autophagy which causes in both cases loss or shorter cilia confirming that autophagy plays a role on ciliogenesis in a context dependent manner. Figure 3 schematizes these conditions which include, to date, focal cortical dyslamination, chronic obstructive pulmonary disease, and thyroid Hurthle cell tumor. We also hypothesize that some of the clinical manifestations observed in ciliopathies, such as renal cystic disease, may be the result of a perturbed autophagy since ciliary proteins and the autophagic machinery are intimately connected.

### 7.1. Focal Cortical Dyslamination

Focal malformations of cortical development (FMCDs) are a heterogeneous group of cortical abnormalities often caused by somatic mutations in mTOR regulatory genes [74,75]. These brain malformations include focal cortical dysplasia (FCD), hemimegalencephaly (HME), and tuberous sclerosis (TSC) [74,76,77].

Park et al. demonstrated that brain somatic mutations in mTOR results in hyper-activation of the pathway and consequent inhibition of autophagy [78]. Furthermore, they showed that in FMCDs neuronal ciliogenesis is defective because of loss of autophagy-mediated degradation of the centriolar satellites portion of the OFD1 protein, a known inhibitor of ciliogenesis [79,80] (Figure 3). These findings were confirmed in murine models, and in brain tissues from HME, FCD, and TSC patients. In addition, the authors demonstrated that disruption of autophagy-mediated ciliogenesis leads to focal cortical dyslamination in FMCDs by abrogating Wnt signaling transduction [78]. 

### 7.2. Chronic Obstructive Pulmonary Disease 

Chronic obstructive pulmonary disease (COPD) is the fourth leading cause of mortality worldwide and results in bronchitis associated with airway inflammation and mucus obstruction, and emphysema caused by chronic cigarette smoke (CS) exposure [81]. Airway epithelial cells have numerous motile cilia specialized into elimination of particles and pathogens from the airways thus acting as a primary innate defense mechanism. CS exposure results in impaired mucociliary clearance attributed to reduced cilia length in epithelial cells and airway epithelial cell death, resulting in excess mucus production which may promote susceptibility to respiratory infections [82,83,84].

It has been shown that autophagy is increased in cultured epithelial cells and in the lungs of COPD patients suggesting that autophagy represents a response to CS exposure, as well as an early event in the progression of emphysema [85]. Lam and colleagues demonstrated that increased autophagy in primary cultured epithelial cells under CS exposure is associated with concomitant cilia shortening and increased amount of ciliary proteins (e.g., Ift88, Arl13, centrin 1, and pericentrin) localized to autophagosome fractions in mouse airways (Figure 3). Moreover, it was shown that genetic inhibition of autophagy exerts a protective effect from CS-induced cilia shortening both in vitro and in vivo [30].

### 7.3. Thyroid Hurthle Cell Tumor

Cilia dysfunction has been proposed as an essential factor in cancer development, however, the role of cilia in tumorigenesis remains elusive [2]. It is well established that increased levels of autophagy promote tumor survival and growth in advanced cancers to overcome stressful conditions, including hypoxia and nutrient deprivation (reviewed in [86]). Ciliogenesis and autophagy are both determining factors in the prognosis of human cancers; however, it is still poorly understood if loss of cilia and upregulated autophagy are associated with cancer [2]. Hurthle cell carcinoma, a thyroid cancer subtype, is an example of cancer showing relatively high basal level of autophagic activity, and concomitantly suppressed ciliogenesis. Lee and colleagues found that both genetic and pharmacologic inhibition of autophagy in Hurthle cell carcinoma restores ciliogenesis and cilia elongation, suggesting that increased autophagy inhibits ciliogenesis in these cells (Figure 3). The authors proposed that defective ciliogenesis could be caused by persistent sequestration of cilioproteins such as IFT88 and ARL13 into autophagosomes [3].

### 7.4. Renal Cystic Disease 

Renal cystic disease (CK) comprises a group of disorders characterized by the development and progressive enlargement of fluid-filled renal cysts that ultimately leads to renal failure. CK is frequently observed in ciliopathies and the commonest condition is represented by autosomal dominant and recessive forms of polycystic kidney disease, ADPKD and ARPKD, respectively. In particular, ADPKD is caused by mutations in *PKD1* and *PKD2*, codifying for PC1 and PC2, which form, in the cilium, an ion channel that allows regulation of calcium release [87,88]. Direct evidence link defects in cilia formation and CK [89,90] including the fact that the majority of cystoproteins, including PC1 and PC2, localize to primary cilia/basal bodies/centrosomes [91]. However, PC1 and PC2 are not critical for cilia formation or stability [91], and cilia are normally present in *Ofd1* inactivated non-cystic renal tubules [92]. Nevertheless, the role of primary cilia in the pathogenesis of renal cystic disease remains unclear. 

mTORC1 is hyperactive in most CK models and in human cysts [92,93,94,95] and has drawn attention for the potential therapeutic use of its inhibitors, rapamycin analogs (rapalogs), which ameliorate the cystic phenotype in CK murine models [92,93,94,95]. Unbalanced autophagy has been associated with renal cystic disease mutants. The importance of basal autophagy as a key homeostatic mechanism to maintain proximal tubules/podocyte integrity has been highlighted [96,97,98]. In addition, autophagy, which is upregulated by stress stimuli such as renal ischemia and nephrotoxins has been described as a surveillance sensor for kidney cells [99]. Decreased autophagy has been described in mutant kidneys of an ARPKD murine model [100], and increased autophagy was shown to improve the renal cystic phenotype in *pkd1* and *pkd2* deficient zebrafish models [101,102]. On the other hand, increased autophagy has been reported in precystic stages in two different models, aqua-porin11 null mice [103] and an ARPKD rat model in which pharmacological inhibition of autophagy significantly reduced cysts growth [104]. 

The experimental evidence on the role of autophagy in PKD remains limited and controversial, despite the recognition of a role for autophagy in the pathogenesis of PKD could be of high clinical relevance. We believe that dissection of the functional interplay between ciliary proteins and autophagy will contribute to elucidate the molecular mechanisms underlying the initial phases of renal cyst formation. This will allow designing therapeutic approaches that could prevent renal cysts formation and/or slow down disease progression.

These examples prompted us to hypothesize that at least some of the clinical manifestations observed in ciliopathies could be due to altered autophagy and thus ciliopathies could be considered as autophagic diseases. On the other hand, we speculate that pathological conditions associated with altered autophagy such as neurodegenerative, muscle, and liver diseases, and cancer could involve abnormal cilia biogenesis and function. 

## 8. Conclusions

Cells adopt a sensory platform, the primary cilium, to control a catabolic process, such as macroautophagy, possibly through the autophagic machinery localized at the peri-ciliary compartment. The cilia-mediated stimuli so far known to act on autophagy are represented by ligands of the Hh family [22], and by bending of the cilium under fluid flow in epithelial cells of renal tubules [42]. We cannot rule out that different stimuli such as light, hormones, insulin, growth factors, could also influence autophagy in a cilium-mediated manner. On the other side, both inhibition and activation of autophagy influence ciliogenesis in a context dependent manner, perhaps to ensure that the sensing organelle is properly formed in a feedback loop model. This intricate relationship explains why the cell needs to compartmentalize autophagic and ciliary proteins in the same cellular district, the peri-ciliary area. 

Numerous proteins localized at cilia and controlling ciliogenesis (under ciliated condition) display a direct functional role in the control of autophagy independently from cilia (in non-ciliated conditions). These findings suggest that, in cycling and sub confluent conditions, these proteins could be considered as novel noncanonical autophagic players, and that, when cells are quiescent and confluent, the same proteins could play both a structural role in cilia, and a regulative role on selective autophagic degradation of positive and/or negative effectors with the final aim of controlling ciliogenesis. However, the identity of the main players, their functional role, the timing of action and the conditions in which the intricate cilia-autophagy crosstalk takes place needs to be established. 

Furthermore, we anticipate that at least some of the clinical manifestations observed in ciliopathies may be the result of perturbed autophagy, considering the cilia-independent role of at least some ciliopathy proteins in autophagy; and, on the other hand we predict that cilia dysfunction could play a role in the increasing number of autophagy-related disorders such as neurodegenerative diseases.

## Figures and Tables

**Table 1:** Proteins involved in the control of both ciliogenesis and autophagy.

Protein	Aliases	Ciliary Localization	MIM#; Phenotype	Effect on Autophagy	Ciliated Conditions	Ref.
IFT20	-	Axoneme	No disease	Interacts with ATG16L and promotes its shuttling from Golgi to cilia; autophagic activity decreased in IFT20-depleted (MEFs and human neuroectodermal) cells	YES	[22,43]
IFT88	D13S1056E, DAF19, TG737, TTC10, hTg737	Axoneme	No disease	Autophagic activity decreased in IFT88-depleted (KECs, HK2, human neuroectodermal) cells	YES	[22,36,42,43]
KIF3A	FLA10, KLP-20	Basal body	No disease	Autophagic activity decreased in KIF3A-silenced human neuroectodermal cells and in murine renal tubular epithelial cells	YES	[42,43]
VPS15	PIK3R4 P150	Axoneme and basal body	cilia phenotype (retinitis pigmentosa, limb abnormalities, renal cysts)	Encodes for VPS34 regulatory subunits. Is involved in autophagosomes formation. Promotes formation and/or release of IFT20 positive vesicles from cis-Golgi to cilia	YES	[44,45,46]
RPGRIP1L	CORS3, FTM, JBTS7, MKS5, NPHP8, PPP1R134	Ciliary transition zone	611560: Joubert syndrome 7 (JBTS7); 611561: Meckel syndrome 5 (MKS5); 216360: COACH syndrome	Autophagic activity decreased in Rpgrip1l deficient MEFs	NO	[29]
INPP5E	CORS1, CPD4, JBTS1, MORMS, PPI5PIV, pharbin	Axoneme	610156: mental retardation, truncal obesity, retinal dystrophy, and micropenis syndrome (MORMS); 213300: Joubert syndrome 1 (JBTS1)	Localizes to lysosomes and is required for autophagosome/lysosome fusion	NO	[47]
PCM1	PTC4, RET/PCM-1	Centriolar satellite	No disease	Interacts with GABARAP and controls its localization and degradation at centriolar satellites thus influencing GABARAP-autophagosome formation	NO	[48]
OCRL	INPP5F, LOCR, NPHL2-1, OCRL-1	Axoneme and basal body	300555: Dent disease-2; 309000: Lowe oculocerebrorenal syndrome	Recruited to lysosomes and required for autophagosome-lysosome fusion	NO	[49]
FLCN	Folliculin BHD FLCL	Axoneme and basal body	135150: Birt-Hogg-Dube syndrome (BHD); 144700: nonpapillary renal carcinoma	Interacts with GABARAP and ULK1 kinase, playing a positive role in autophagy. Involved in signaling amino acid levels to mTOR kinase at lysosomes	NO	[50,51]
HTT	Huntingtin HD Protein	Basal body	143100: Huntington disease	Interacts with p62 and ULK1 kinase; required for selective autophagy	NO	[52]
